# Multi-End Physics-Informed Deep Learning for Seismic Response Estimation

**DOI:** 10.3390/s22103697

**Published:** 2022-05-12

**Authors:** Peng Ni, Limin Sun, Jipeng Yang, Yixian Li

**Affiliations:** 1 Department of Bridge Engineering, College of Civil Engineering, Tongji University, Shanghai 200092, China; 2032517@tongji.edu.cn (P.N.); lmsun@tongji.edu.cn (L.S.); yangjplut@163.com (J.Y.); 2 State Key Laboratory of Disaster Reduction in Civil Engineering, Department of Bridge Engineering, College of Civil Engineering, Tongji University, Shanghai 200092, China; 3 Department of Civil and Environmental Engineering, The Hong Kong Polytechnic University, Hong Kong

**Keywords:** physics-informed neural network, multi-end autoencoder, seismic response reconstruction, data conversion, structural health monitoring

## Abstract

As a structural health monitoring (SHM) system can hardly measure all the needed responses, estimating the target response from the measured responses has become an important task. Deep neural networks (NNs) have a strong nonlinear mapping ability, and they are widely used in response reconstruction works. The mapping relation among different responses is learned by a NN given a large training set. In some cases, however, especially for rare events such as earthquakes, it is difficult to obtain a large training dataset. This paper used a convolution NN to reconstruct structure response under rare events with small datasets, and the main innovations include two aspects. Firstly, we proposed a multi-end autoencoder architecture with skip connections, which compresses the parameter space, to estimate the unmeasured responses. It extracts the shared patterns in the encoder and reconstructs different types of target responses in varied branches of the decoder. Secondly, the physics-based loss function, derived from the dynamic equilibrium equation, was adopted to guide the training direction and suppress the overfitting effect. The proposed NN takes the acceleration at limited positions as input. The output is the displacement, velocity, and acceleration responses at all positions. Two numerical studies validated that the proposed framework applies to both linear and nonlinear systems. The physics-informed NN had a higher performance than the ordinary NN with small datasets, especially when the training data contained noise.

## 1. Introduction

The past decades have witnessed rapid development and many applications of structural health monitoring (SHM) systems [1]. Many buildings [2] have been equipped with the SHM system to monitor their real-time responses and evaluate their working conditions [3,4]. For civil structures, the seismic responses are essential to evaluate their health and working conditions [5,6]. However, measuring all desired responses requires too much effort, and it is usually not economic. Data conversion algorithms that convert the measured data to the desired responses have emerged to address this issue.

Current approaches can be separated into model-driven and data-driven approaches. The model-driven methods principally rely on prior knowledge about the studied system, and in most cases, a mechanical model or finite element model (FEM) is a required known [7]. This means the modeling error affects the algorithm performance except for some special cases [8,9]. On the other hand, the data-driven methods directly utilize the mapping relations among different responses to calculate the target responses from the easy-measuring responses [10]. With the maturity of deep learning, deep neural networks (DNNs) have been used in response reconstruction works, such as lost data recovery in which the DNN learns the mapping relationship among sensors to recover the lost data from the operating sensors [11,12,13,14].

Since traditional deep learning in a supervised manner requires a large dataset that is difficult or costly to collect, it is meaningful to train a DNN with a small dataset. Considering that the physical laws can provide additional constraints to the deep learning problem, physics-informed deep learning (PIDL, also named physics-guided or physics-constrained deep learning) has recently emerged in many areas where it takes much effort to collect a large dataset, such as computational fluid dynamics [15] and corrosion-fatigue analysis [16]. PIDL can reduce the dataset volume and simultaneously improve accuracy compared with the ordinary deep learning approaches.

The PIDL has developed along two routes: theoretical evolution and engineering applications. The partial differential equation (PDE) is the governing equation for many problems. The original theoretical research of PIDL is for solving PDEs using fewer [17,18] or no [15,19,20,21] training datasets, given different initial and boundary conditions. Among them, the loss function is composed of the governing PDE, initial condition, and boundary condition, for a training data-free solution:

(1) $$L=\alpha_{D}MSE_{D}+\alpha_{I}MSE_{I}+\alpha_{B}MSE_{B}$$

where *L* represents the loss function. *MSE* refers to the mean square error. *α* is constant weighting coefficients. The subscripts *D*, *I*, and *B* indicate the differential equation, initial condition, and boundary condition. If the training data are involved, the loss function becomes [22]:

(2) $$L=\alpha_{T}MSE_{T}+\alpha_{D}MSE_{D}+\alpha_{I}MSE_{I}+\alpha_{B}MSE_{B}$$

where the subscript *T* refers to the training set.

When the training loss reduces, the trained network always approaches the near-optimal solution of a PDE. Inspired by this, PIDL has been extended to engineering applications. The related studies have involved many communities, including fluid dynamics [15,23,24], geology [25], fatigue analysis [26], power system [27], and system identification [28] and controls [29]. In PIDL, a conventional strategy is writing the governing PDE into the loss function [30] to compress the solution space. It is equivalent to adding a regularization term. Moreover, the network architectures can be designed considering the focused problems. This means we can mimic the underlying physical phenomenon using a DNN [31] or use it describe some ambiguous physical laws [16,26,29], and even combine some physics-guided computations in a NN [27]. Current PIDL studies are summarized in Table 1. However, the application of PIDL in SHM is still rare [32], considering deep learning is widely used in the SHM community.

Furthermore, the approach becomes semi-supervised when the loss function is formed as Equation (2). Self-supervised deep learning requires labeled training data (or the ground truth) to calculate the empirical loss $MSE_{T}$, and attempts to minimize it during the learning process. The added physical loss can help guide training without ground truth. The concept and method of applying semi-supervised learning for fully convolutional networks are introduced in detail in [35]. Since it can reduce the effort to prepare the training samples, semi-supervised learning is adopted in many engineering applications, such as building detection [16] and remaining life prognostics [36,37].

This study proposed a multi-end convolutional neural network aiming to reconstruct the full DOF seismic responses of a structure. Based on the measured ground motion and accelerations at limited DOFs of a frame, the proposed approach could provide multi-types of responses at all DOFs. Furthermore, inspired by the PIDL, physics-based prior knowledge was incorporated into the network by transforming it into an additional loss item. Since obtaining a great volume of measured data for earthquake events is difficult or even impossible, the introduced physical loss function could help improve the performance of the network with a small training dataset. The contributions of the paper can be summarized as follows:

- We proposed a convolutional network with an autoencoder architecture and multi-ends. With this structure, the network could fuse the input data together and reconstruct various types of responses at all the DOFs.
- We showed that the performance of the network on small datasets could be improved by introducing a physical loss function.
- The proposed network model had a strong fitting ability and stable accuracy in both linear and nonlinear numerical examples.

This paper is arranged as follows: Section 2 introduces the methodology, including the physics-informed loss function, the multi-end network architecture, and the combination mode of them. Furthermore, the data structure and optimization plan, Adam, is also explained. Section 3 and Section 4 are two numerical studies. In Section 3, we validate the performance of the proposed approach on a linear system, where the detailed algorithm procedure is introduced. The hyperparameter and sensitivity analysis is also introduced. Then, to show the strong ability of the proposed network model, we further apply it to a nonlinear system in Section 4. The final section, Section 5, draws the main conclusions.

## 2. Methodology

This section proposes a multi-end physics-informed neural network (PINN) to predict the seismic response of the multi-DOF structures. The innovation of the proposed network principally includes two aspects: the multi-end architecture and the physics-guided loss. The adopted network is composed of convolutional layers without pooling and has an autoencoder architecture. In the encoder, the origin measurement is gradually compressed as higher-level patterns. After that, three decoders can independently reconstruct displacement, velocity, and acceleration responses at all DOFs. In addition, skip connections are used to transmit the low-level patterns in the shallow layers to the decoder to prevent the loss of information at the bottleneck layer. The section details the design and arrangement of the proposed network.

### 2.1. Multi-End Convolutional Network

#### 2.1.1. Convolution Layers

The convolutional neural network was adopted in this paper due to its superior performance in processing structured data and time series, and the monitored data was time-synchronized and structured. Firstly, the computing principle of a convolution layer is briefly introduced here.

**Figure 1:**
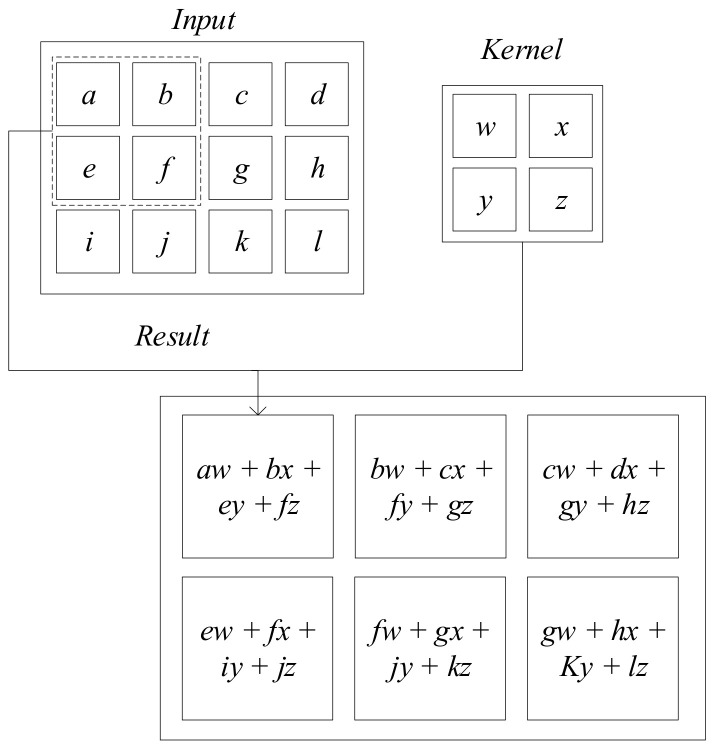
Graphic explanation of 2-D convolution [38].

Consider the monitored data as a 2-dimensional input tensor (*I*). The computing principle of the 2-D convolution with a kernel *K* is:

(3) $$S(i,j)=(I*K)(i,j)=\sum_{w}\sum_{h}I(i+w,j+h)K(w,h)+b$$

where *i* and *j* are the element positioning indexes of the resultant array *S*. *w* and *h* are the width and height indexes of kernel *K*, respectively. *b* is a constant bias term. The graphic explanation is shown in Figure 1. The elements of a single kernel are identical for the entire input domain, which enhances the parameter-sharing effect and reduces the total parameter number compared with the densely connected layers.

Similar to a grayscale picture, the monitored data were a 2-D tensor, whereas its width (data length) was far greater than its height (sensor number). The kernel size became an important hyperparameter in this study, where the kernel width controlled the receptive field, and the height determined the data fusion pattern. The selection of kernel height was relatively simpler. If the kernel height was one, the monitored data were independently used in the convolutional layer to calculate the resultant tensor; if the kernel height was greater than one, the convolutional layers had some data-fusion characteristics. The kernel width was more significant in this paper since it determined the receptive width. A greater kernel width means the kernel can “scan” a longer period of information. Furthermore, the deeper layers inherently have a greater receptive field since they directly take the former layer-computed result as their input. This means the shallow layers can extract the short-period information and the deep layers can extract the long-period information.

The convolution calculation in Figure 1 is linear because the resultant matrix was linearly computed (multiplying and adding) by the kernel and input. To enhance its nonlinear mapping ability, an activation function was necessary, with which the elements in the resultant matrix become:

(4) $${S(i,j)}^{\prime }=a[S(i,j)]$$

where *a* is the activation function. The most widely used activation functions include the sigmoid, tanh, rectified, and leaky rectified functions. In this paper, the rectified linear unit (Relu) was adopted as the activation function. A (leaky) Relu has the following form:

(5) $$a(x)=\left\{\begin{array}{ll} x & \left(x>0\right)\\ kx & \left(x\leq 0\right) \end{array}\right.$$

where *x* is the input value and *k* is a constant (*k* ≥ 0). The function is named Relu when *k* = 0, and named leaky Relu when *k* is a small positive number. In this paper, Relu activation was selected.

#### 2.1.2. Skip Connection

**Figure 2:**
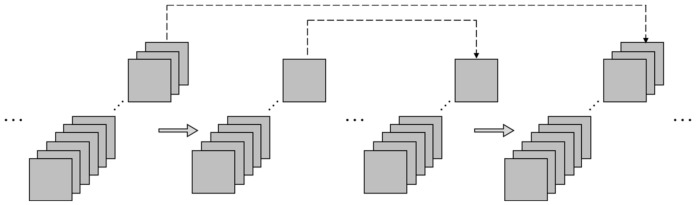
Skip connection.

Skip connection is initially used for addressing the gradient vanishing problem when a network is too deep and the gradient can be near zero after multi-layers of backpropagation. With the skip connections, the shallow layers are directly connected to the deeper layers in a skipping manner, rather than the traditional layer-by-layer transforming manner. Therefore, the gradient in the deeper layers can be backpropagated to the shallow layers to address the gradient vanishing problem. In addition, the shallow layer-extracted information is also delivered to the deep layers in the forward propagation (see Figure 2).

**Figure 3:**
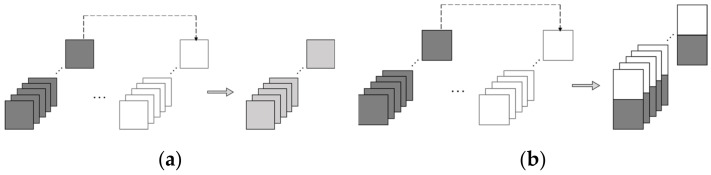
Two skip modes.

The skip connection is especially beneficial in an autoencoder structure because the low-level (short-period) information cannot all be retained in the bottleneck layer. A skip connection can help the decoder to utilize the encoder-extracted information, rather than only the bottleneck layer-retained information, when predicting the target outputs. The shallow layers’ output can skippingly propagate to the deeper layers in two modes: the adding or concatenating modes. The adding skip connection (see Figure 3a) sums the resultant tensors of two layers up, which requires the tensor shapes to be identical. The concatenating skip connection stacks the output tensors from two layers to form an extended tensor (see Figure 3b). In this paper, the adding skip was adopted as it can better “merge” the data and keep the tensor shape unchanged.

#### 2.1.3. Multi-End Neural Network Architecture

**Figure 4:**
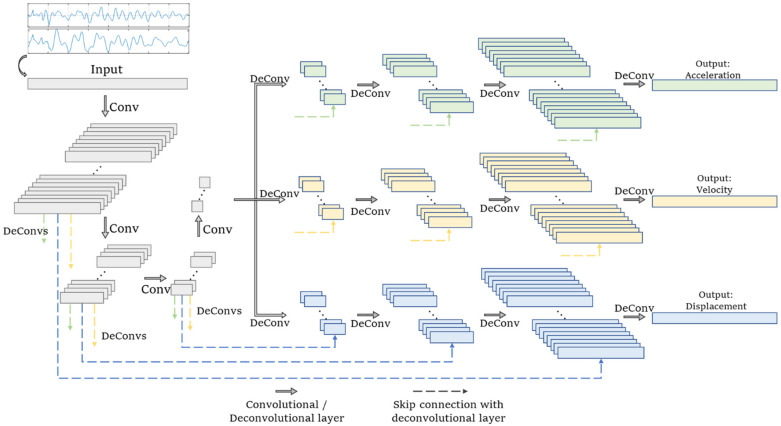
Network structure.

There are many different responses, such as displacement, velocity, acceleration, inclination, strain, etc. They have varied characteristics and modes. For example, acceleration has more high-frequency components, while strain or displacement contains more low-frequency information. The mappings from the monitored data to different types of responses are varied. When the output of a single NN involves several response types, the NN has to learn several different mapping modes, which implies it needs more layers and kernels to provide that all mappings are well-learned. In this case, the parameter number is over-large and the overfitting effect becomes severer. To balance the training efficiency, overfitting effect, and accuracy, this study adopted a multi-end autoencoder architecture (see Figure 4) to reconstruct the target responses.

The synchronized monitoring data form a 2-D input array. Its height corresponds to the sensor number, and the width is the length of the data piece. The network output has three branches that aim to reconstruct varied types of responses. The output of each branch is a 2-D array. Its width is the data length, and its height is determined by the number of target responses. The network only involves convolutional and deconvolutional layers without pooling. Skip connections are designed with which the convolutional layers in the encoder are directly connected to all three decoder branches. Due to the limitation of in-plane plotting, only the skip connections to the third branch are completely illustrated.

In the encoder, the original information is gradually compressed. After each layer, the width of data is halved, and the kernel number is halved as well. The first convolutional layer has 128 kernels, and three layers later, the kernel number is reduced to 16 (=128/2^3^) in the bottleneck layer. With the decrease in tensor width and kernel number, the tensor height remains unchanged. As the sensor number is relatively small, compressing the data height means some detailed information is abandoned, and thus, the tensor height is not compressed in the encoder. The encoder converts the 2-D tensor of input into 3-D tensors of the hidden layers (the third dimension corresponds to the data depth) that contain data patterns. The 3-D tensors after the shallow convolutional layers retain the low-level patterns, while tensors in the deeper layers correspond to the high-level patterns because the deep encoder layers inherently have a greater receptive field.

In the decoder, the extracted latent variables are gradually transformed into three types of responses: acceleration, velocity, and displacement. The network in Figure 4 has three branches that individually correspond to three response types, and there are no connections among the three branches. Take the branch for reconstructing the acceleration as an example. It has four deconvolutional layers that convert the latent variables in the bottleneck to the target. There are also skip connections containing both a deconvolutional layer and an adding layer, which convert the encoder-extracted patterns to the target responses. Skip connections provide the completeness of the information. The decoder branches for reconstructing the velocity and displacement have identical architectures and are not detailed again.

### 2.2. Physics-Informed Loss Function

As mentioned in the introduction, big data is the basis of deep learning. In some scenarios, the training data are extremely limited. This study focused on the seismic response reconstruction problem where the collected training data are rare. To avoid the overfitting effect, a physics-based regularization term was added.

The physical losses include the differential equation, initial condition, and boundary condition losses (see Equation (1)). In SHM, the initial and boundary conditions, which are both zeros, are relatively simpler than those in the fluid problems. The governing equations of most problems are partial differential equations:

(6) $$u(t)=N(t,x,u,u_{x},u_{t},u_{xx},u_{tt}...)$$

where *u*, *t*, and *x* represent the solution (resultant state), time, and solution space, respectively. N refers to a nonlinear function. The subscript denotes the partial differentiation with respect to either time *t* or space *x*.

The governing differential equation for a space-discrete mechanical system is not a partial differentiation because the differentiation with respect to space *x* is avoided, and only the differentiation over time is retained:

(7) $$u=N(u,u_{t},t_{tt})$$

The relationship in Equation (7) can form the physical loss function:

(8) $$L_{p}=\left|u-N(u,u_{t},t_{tt})\right|$$

Considering that the most widely used physical law in structural analysis is the dynamic equilibrium equation in the space-discrete form, the physical loss function can be written as:

(9) $$L_{p}=\left|M\overset{\cdot \cdot }{u}+C(t)\overset{\cdot }{u}+K(t)u-F(t)\right|$$

where **M**, **C**(*t*), and **K**(*t*) $\in {\mathbb{R}}^{n\times n}$ denote the mass, damping, and stiffness matrices that are assumed known (they can be time-varying or invariant), respectively. **F**$\in {\mathbb{R}}^{n\times 1}$ is the external force vector, and **u**$\in {\mathbb{R}}^{n\times 1}$ is the displacement vector. *n* is an integer denoting the degrees of freedom (DOFs).

The dynamic equilibrium equation must be satisfied at each moment for both linear and nonlinear systems. Therefore, the physical loss Equation (9) can provide an additional constraint to the estimated responses to prevent the training process from overfitting on the training set. In Equation (9), the loss function involves known acceleration, velocity, displacement, and external force **F**. However, the external input is rarely known for SHM since it contains complex components, including the wind, humans, temperature, ground motion, etc. Fortunately, the external load can be regarded as known during an earthquake. Considering the motion of a multi-DOF frame, the equilibrium equation is:

(10) $$M\overset{\cdot \cdot }{u}+C(t)\overset{\cdot }{u}+K(t)u=-M1{\overset{\cdot \cdot }{u}}_{g}\equiv F(t)$$

where *u_g_* denotes the ground motion and **1**$\in {\mathbb{R}}^{n\times 1}$ is a vector in which elements are all equal to one. When the ground acceleration ${\ddot{u}}_{g}$ is monitored, the equivalent external load **F** is known as well. In this case, the dynamic equilibrium Equation (10) can provide an additional constraint. In other loading cases, the external force **F** is difficult to directly measure.

The key step of physics-informed deep learning is designing the loss function. This study falls into the supervised deep learning category, and therefore, the loss function includes two parts. The first part is the training loss *L_T_*:

(11) $$L_{T}=\frac{1}{N}\sum_{i=1}^{E}\sum_{j=1}^{T}\sum_{k=1}^{N}\alpha_{i}{\left(y_{i,j,k}-{\hat{y}}_{i,j,k}\right)}^{T}(y_{i,j,k}-{\hat{y}}_{i,j,k})$$

where **y** and $\hat{y}$ are the ground truth and reconstructed response vectors. *i* indicates the branch number (*i* = 1~3 and *E* = 3). *j* indicates the output number in each branch. *k* is the sample number index and *N* is the total sample number. *E* represents the total number of data types and *T* denotes the length of data series for each type. The magnitudes of displacement, velocity, and acceleration deviate a great deal from each other, and therefore, constant coefficients $\alpha_{i}$ are multiplied to provide the output magnitudes of all branches near each other.

The physical loss that is determined by the dynamic equilibrium equation was considered as well. With Equation (9), the residual force can formulate the physical loss function that has the following form on the whole training set:

(12) $$L_{P}=\frac{1}{N}\sum_{l=1}^{W}\sum_{k=1}^{N}{\left[M{\overset{\cdot \cdot }{u}}_{l,k}+C{\overset{\cdot }{u}}_{l,k}+Ku_{l,k}+M1{\left({\overset{\cdot \cdot }{u}}_{g}\right)}_{l,k}\right]}^{T}[M{\overset{\cdot \cdot }{u}}_{l,k}+C{\overset{\cdot }{u}}_{l,k}+Ku_{l,k}+M1{\left({\overset{\cdot \cdot }{u}}_{g}\right)}_{l,k}]$$

where *l* indicates the sampling moment. The output width is *W*. **u** is the reconstructed displacement. In Equation (12), $\ddot{u}$, $\;\dot{u}$, and **u** are outputs of the network. The ground acceleration ${\ddot{u}}_{g}$ is directly measured.

**Figure 5:**
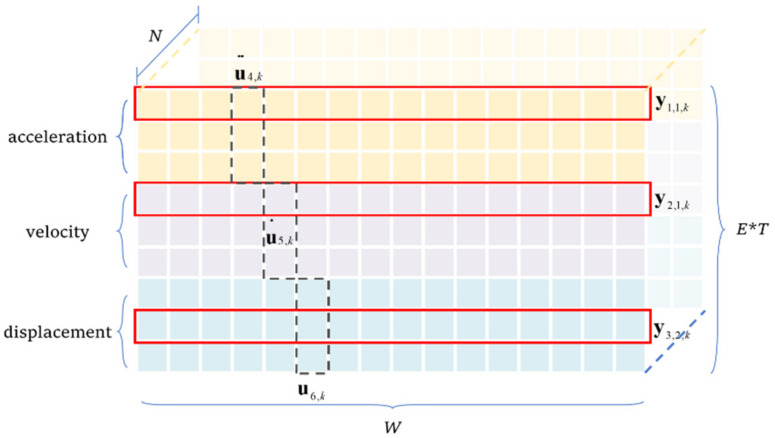
Data structure of the output.

Figure 5 shows the output data structure of one training sample. The rows correspond to y and $\hat{y}$ in Equation (11). The columns are the acceleration, velocity, and displacement vectors at each sampling moment in Equation (12).

The loss function is the weighted sum of the training and physical losses:

(13) $$J=L_{T}+\beta L_{P}$$

where β is an important constant hyperparameter. In a training, β should be carefully tested and selected. For different structures, the residual force in Equation (12) has different magnitudes and values. β must provide that the weights of the training loss and physical loss coordinate. If the weight of the physical loss is over-large, it suppresses the reconstructed response magnitude because the physical loss is minimum with all responses being zero. When β is too minor, the physical loss has little contribution to the whole loss function and the result is not be modified. Therefore, the determination of β needs a fine-tuning process. In this paper, β was selected in a trial method after many iterations of numerical tests.

With the Equation (13)-defined loss function, the optimization problem becomes:

(14) $$\begin{array}{ll} \underset{\left(W\right)}{\min}J(W)=\frac{1}{N}\sum_{i=1}^{E}\sum_{j=1}^{T}\sum_{k=1}^{N}\alpha_{i}{\left(y_{i,j,k}-{\hat{y}}_{i,j,k\left|W\right.}\right)}^{T}(y_{i,j,k}-{\hat{y}}_{i,j,k\left|W\right.})+ & \\ \frac{\beta }{N}\sum_{l=1}^{W}\sum_{k=1}^{N}{\left[M{\overset{\cdot \cdot }{u}}_{l,k\left|W\right.}+C{\overset{\cdot }{u}}_{l,k\left|W\right.}+Ku_{l,k\left|W\right.}+M1{\left({\overset{\cdot \cdot }{u}}_{g}\right)}_{l,k}\right]}^{T}[M{\overset{\cdot \cdot }{u}}_{l,k\left|W\right.}+ & C{\overset{\cdot }{u}}_{l,k\left|W\right.}+Ku_{l,k\left|W\right.}+M1{\left({\overset{\cdot \cdot }{u}}_{g}\right)}_{l,k}] \end{array}$$

where **W** is the parameter space of the network. The “|**W**” in the subscript indicates that the corresponding vectors are calculated by the PINN given a group of parameters **W**. The training of the PINN is equivalent to finding the optimal parameters **W** to minimize the loss function *J*.

### 2.3. Training Details

The proposed PINN is a supervised learning task, and its training process is similar to the traditional DNN. The input data is fed into the network to predict the target response, and the difference between the ground truth and the prediction comprises the training loss. The network parameters **W** are gradually modified by a gradient-based backpropagation algorithm to minimize the loss function. This section briefs some essential details of the training process.

(1) Training data preparation

This paper adopted piece-wise data to form the input and output pairs for training. First, all monitored seismic response records were connected to obtain an overall record, and then, the connected record was broken into pieces with a certain length to generate the training samples. The samples had intersections with each other to increase the sample number. As displacement, velocity, and acceleration have different magnitudes, they were normalized by the factors α_i_ in Equation (11) to provide that they have similar weights in the training loss *L_T_*. However, the physical loss requires their magnitudes to remain unchanged, and they were not normalized in *L_P_*.

In the numerical simulation of this paper, six seismic waves were used to generate the training set. The connected response data were broken into pieces to a length of 400 to get the training samples. The network input involved the structural acceleration at two DOFs and the ground acceleration. The output was the acceleration, velocity, and displacement responses at all DOFs.

(2) Training process

The optimizing algorithm and learning rate should balance the training efficiency and accuracy. When minimizing the loss function in Equation (14), the network parameters W were updated based on the calculated gradient ∂J/∂W. To stabilize and accelerate this process, the adaptive moments (Adam) algorithm was adopted in this paper. Adam computes an exponentially weighted average of the gradient and it can retain the training momentum and direction in the former steps. The algorithm detail is explained in Algorithm 1.
**Algorithm 1**: Adam optimizer [38]**Require**: Global learning rate ϵ, decay rate ρ**Require**: Initial parameter **W****Require**: A small constant δ to avoid division by zero Initialize accumulation variable **r = 0** **while** stopping criterion not met **do**  Sample a minibatch with *m* samples $\left\{x^{\left(1\right)},...,x^{\left(m\right)}\right\}$ from the training set with corresponding targets $\left\{y^{\left(1\right)},...,y^{\left(m\right)}\right\}$  Compute gradient: $g\;=\;\frac{1}{m}\nabla_{W}(\overset{m}{\underset{i=1}{\sum }}J_{i})$   Accumulate squared gradient: **r** = ρr + (1 - ρ)g⊙g             (15)  (⊙ represents the element-wise multiplying)   Parameter updating: $W\;=\;W\;-\;\frac{\epsilon }{\sqrt{r}+\delta }⊙g$                  (16)  (root square and division are all conducted element-wise)**end while**

**Figure 6:**
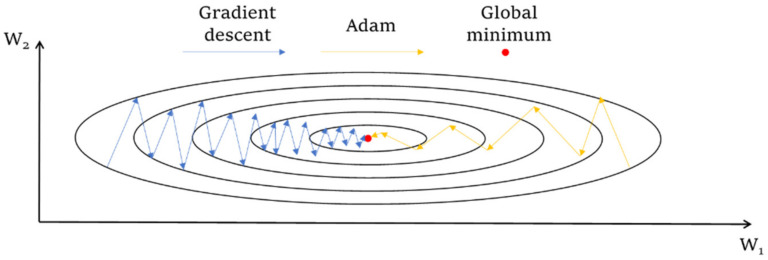
Effect of the Adam optimizer.

Figure 6 illustrates an intuitive explanation of Adam, where only two parameters W_1_ and W_2_ are considered. The gradient in the W_1_ direction is gentle while that in W_2_ is steep. One wants to accelerate the descending speed in W_1_ but slow down that in W_2_, without any prior information about their gradient distribution, and cannot assign varied learning rates in these two directions. The Adam optimizer achieves this aim using Equations (15) and (16). The decaying rate ρ retains the former descending trend: the moving direction in W_1_ is consistent in the whole training process, and such a moving trend is well-retained; moving in the W_2_ direction is unstable, and Adam stabilizes it by considering the former moving trend with ρ. The other metric of the Adam optimizer is the square and root-square operators over gradient **g** in Equations (15) and (16). The gradient in W_1_ direction is minor, and the corresponding term $\epsilon /(\sqrt{r}\;+\;\delta )$ is greater to accelerate the descending speed. The function of $\epsilon /(\sqrt{r}\;+\;\delta )$ is opposite in the W_2_ direction. In summary, the Adam adopts two strategies simultaneously to accelerate and stabilize the gradient descending process, and it is a superior methodology.

## 3. Numerical Study on a Linear System

**Figure 7:**
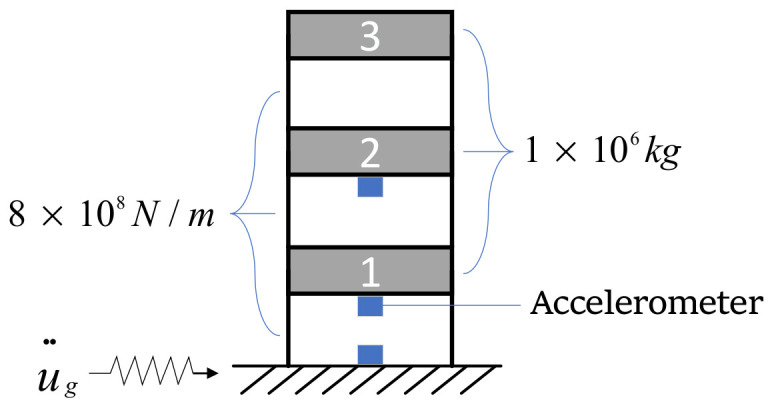
Model illustration.

This section uses a linear multi-DOF system to validate the proposed PINN in response reconstruction. The model is a three-story frame with identical story stiffnesses and masses (see Figure 7). As the model is simple, the mass, damping, and stiffness matrices were computed directly without using a finite element software:

(17) M=1 × 1060001 × 106000 1 × 106 C=2.096 × 1064.582 × 10404.582 × 1042.096 × 1064.582 × 10404.582 × 1042.096 × 106 K=1.6 × 109−8 × 1080−8 × 1081.6 × 109−8 × 1080−8 × 1081.6 × 109 

The natural frequencies were 2.0034, 5.6134, and 8.1116 Hz. The first two modal damping ratios were 2% and 3% where the Rayleigh damping was adopted. The responses were computed using the Newmark-Beta method with a computing frequency of 100 Hz.

**Figure 8:**
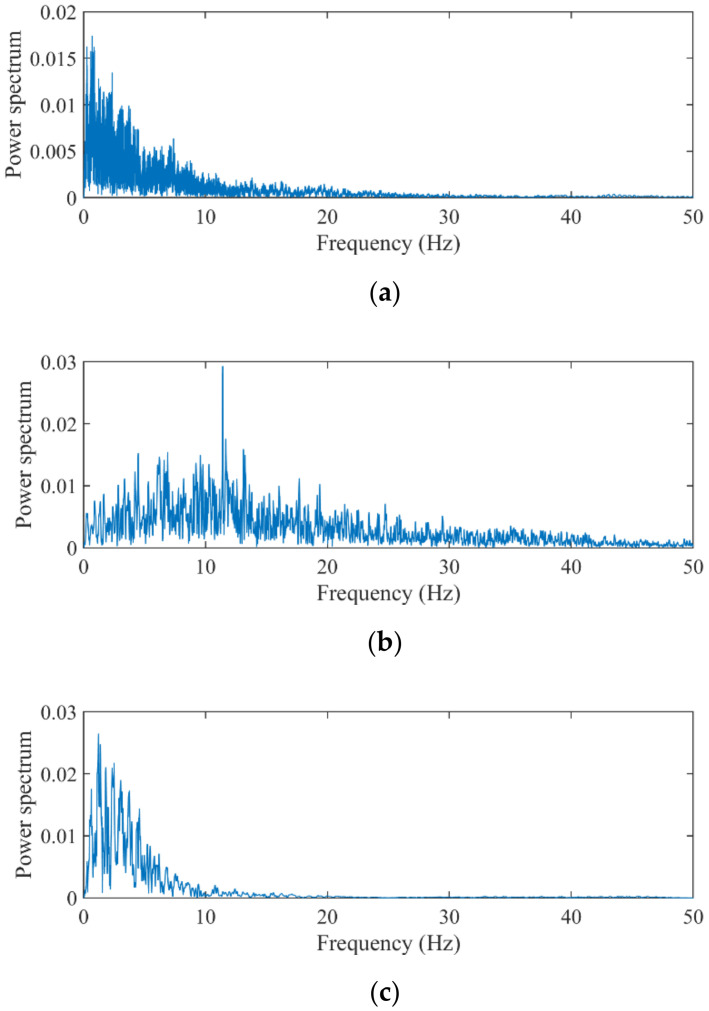
Spectrum distributions of the seismic waves. (**a**) Training set: first to sixth waves; (**b**) testing set: Kocaeli wave; and (**c**) testing set: Landers wave.

Eight earthquake waves were downloaded from the “Pacific earthquake engineering research center”, and their summary is detailed in Table 2. The sampling and computational frequencies were 100 Hz. The frame responses under the first six waves formed the training set. The testing set was the responses under the remaining two waves. The frequency ranges of all waves covered the numerical model’s natural frequencies. The training set (0.02–50 Hz) covered the 7th wave in the frequency spectrum but not the 8th. The spectrum power of the training set concentrated below 10 Hz (see Figure 8). Compared with the Kocaeli wave, the landers wave shared more similarities with the training set in the frequency spectrum. This small difference caused a minor variation in the algorithmic performance on the testing set.

### 3.1. Training and Testing the PINN

The first step was to prepare the training set, i.e., calculating the displacement, velocity, and acceleration response of the frame under the first six seismic waves. Note that the resultant force in Equation (9) must be close to zero to provide that the physical loss can correctly guide the training direction. Then, the calculated responses were stacked vertically to form a matrix, in which the columns and rows were identical to Figure 5. This paper reconstructed the seismic response in a piece-wise manner. The training samples were generated by a moving window with a length of 400 (4 s) and a moving step of 50 (0.5 s). There were a total of 481 training samples. The network input was the accelerations at the DOF 1 and 2, as well as the ground acceleration (see Figure 7). The output was the displacement, velocity, and acceleration responses at all DOFs.

The network architecture is already illustrated in Figure 4. Here, the hyperparameters are detailed in Table 3. There were a total of 649,783 trainable parameters, far greater than the sample number of 481. Though the decoder has three branches, they have an identical architecture and only the hyperparameters of one branch are given. The network input shape was 3 × 400. The output shape of each branch was 3 × 400, and the overall output shape was 9 × 400.

**Figure 9:**
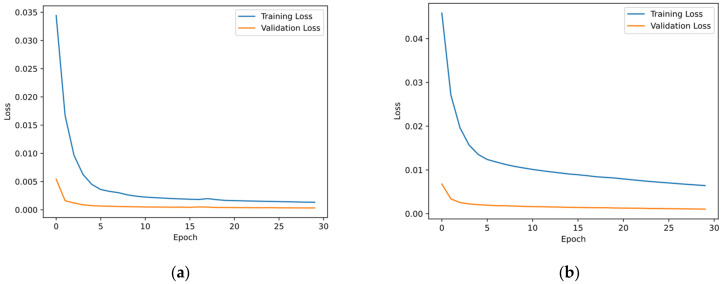
Comparison of the training process of the two networks. (**a**) Without physical loss; (**b**) with physical loss. As mentioned before, the hyperparameter *β* should be selected carefully.

The computer configuration for training included: the CPU processor Intel(R) Core (TM) i7-10875 @ 2.30Hz, and the GPU graphic hardware NVIDIA 2080s. The network was trained by 30 epochs with a batch size of 32. The training time for each epoch was 3.26 s and the total training time was 98s, in which the physical loss did not increase the training time. The losses of an ordinary NN and a physics-informed NN are compared in Figure 9. The losses in (a) descended quickly. After ten epochs, the training loss reduced below 0.002 and did not furtherly descend, which indicated that overfitting appeared. The training loss of the PINN in (b) was greater than (a) since the loss function involved the physical loss term, and it could not descend to the same level as (a). In addition, the training loss here was greater than the validation loss because of two reasons. Firstly, the validation ratio was extremely small and the validation dataset could have responses near zero. Secondly, the validation overlapped with the training set since the samples were obtained using a moving window.

**Figure 10:**
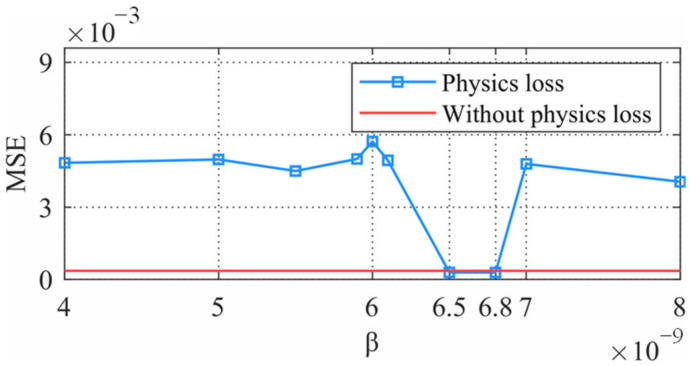
Network performance with different *β*.

Figure 10 shows the MSE of the reconstructed displacement at DOF 2 with different *β* values. The performance of the network was sensitive to the value of *β*. Therefore, training with various *β* values and finding an optimal value is an important task. When *β* was between 6.5 to 6.8 × 10^−9^, the PINN had higher accuracy than the CNN. Herein, we selected the value of *β* to be 6.5 × 10^−9^.

**Figure 11:**
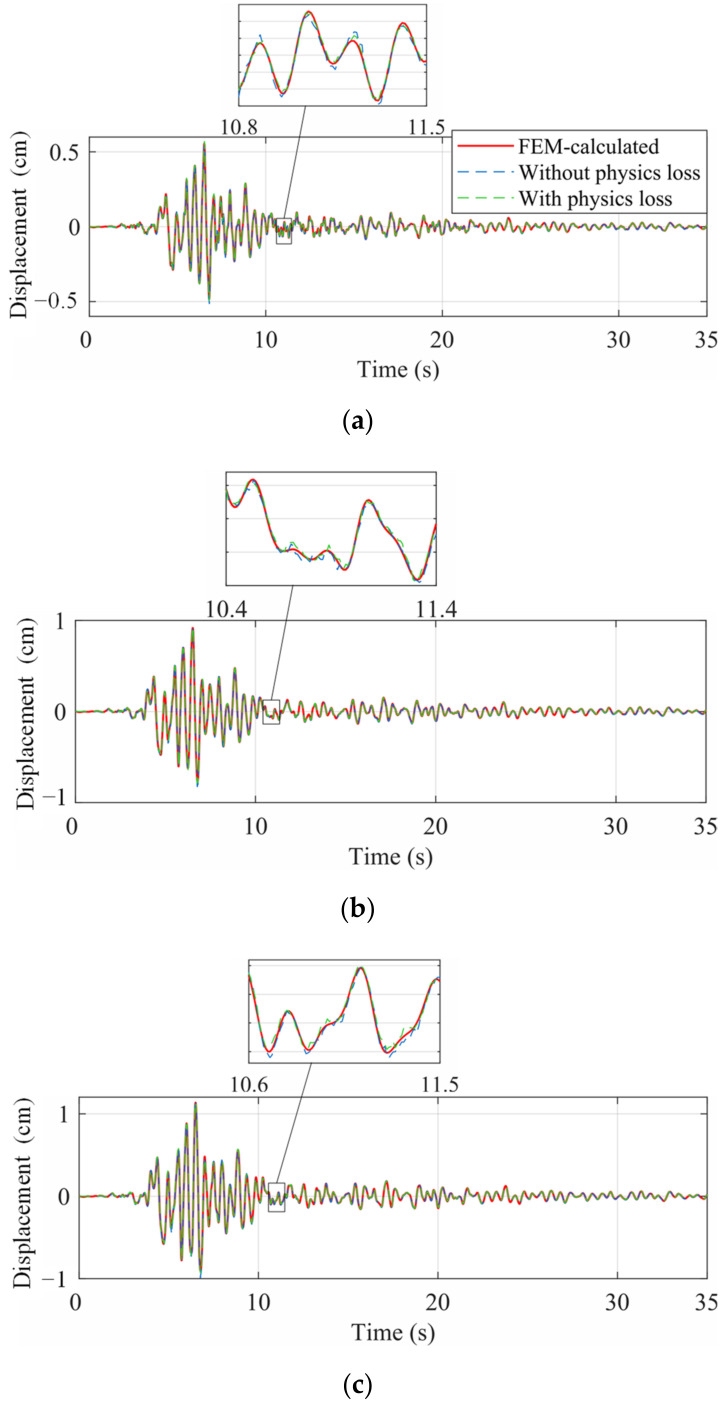
Reconstructed displacement under the Landers wave. (**a**) DOF1; (**b**) DOF2; and (**c**) DOF3.

**Figure 12:**
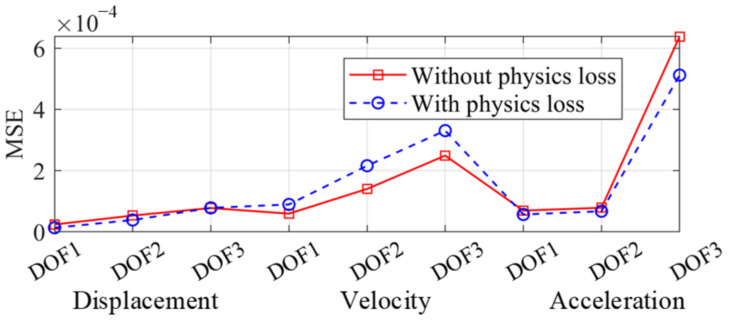
MSE distribution (Landers wave).

The seismic response of the frame was reconstructed by the two trained networks, independently. The spectrum of the Landers wave was more similar to the training set and the corresponding frame response was firstly reconstructed in Figure 11. The reconstructed responses of the ordinary NN and the PINN were both accurate and close to the real response, which indicated that the proposed multi-end architecture could predict the seismic response of a frame given a small training dataset, even though overfitting existed. The mean square errors (MSE) of the two networks are shown in Figure 12. The MSEs of displacement and acceleration were lower adopting the PINN, while the MSE of velocity was greater. This is because in the dynamic equilibrium equation, the inertial and elastic-restoring forces have greater amplitudes, and the acceleration and displacement are given greater weights. Therefore, the accuracy of the reconstructed velocity is sacrificed in the physical loss. As displacement and acceleration are more significant when evaluating the structure performance, the inaccuracy of the velocity is acceptable.

**Figure 13:**
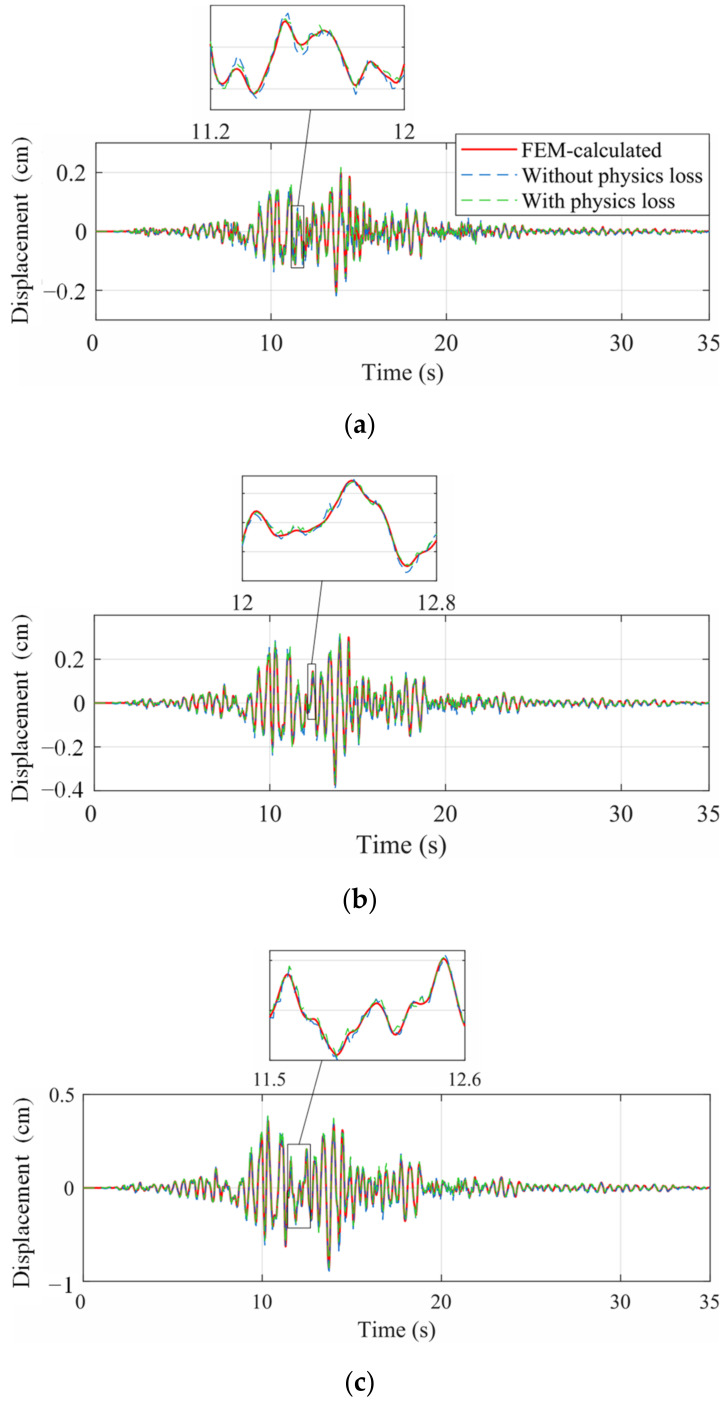
Reconstructed displacement under the Kocaeli wave. (**a**) DOF1; (**b**) DOF2; and (**c**) DOF3.

**Figure 14:**
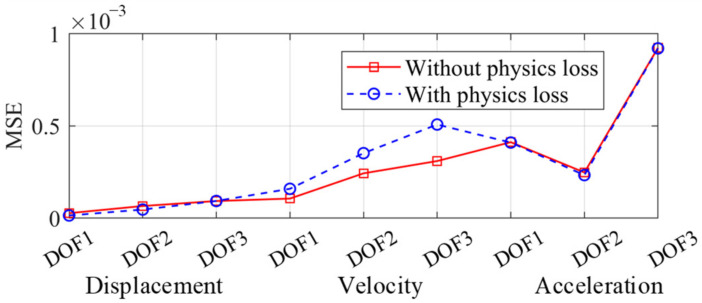
MSE distribution under the Kocaeli wave.

The frame responses under the Kocaeli wave were reconstructed as well (see Figure 13). The MSEs are shown in Figure 14, and the error level was greater than Figure 12 because the spectrum of the training set was more similar to the Landers wave. Even so, the reconstructed responses still accorded well with the real responses. Under the Kocaeli wave, the reconstructed displacement by the PINN was more accurate, while the velocity had more errors (see Figure 14).

In the testing set, the physical loss reduced the MSEs of the reconstructed displacement and acceleration. However, the physical loss had a positive function only when the ordinary NN-estimated responses were accurate enough, where the physical loss could modify the responses. Namely, merely introducing a physical loss could not fundamentally improve the network’s fitting ability. If the estimated responses deviated too far from the real responses, the physical loss could further reduce the accuracy. Therefore, an appropriate DNN architecture is the basis of the PINN.

### 3.2. Data Source

The sensor type and sensor arrangement can also influence the accuracy of the proposed framework. In the former sections, the adopted sensor involved only accelerometers. In practice, the velocimeter and displacement transducers are also widely used. This section shortly discusses the performance of the PINN with different measurements as its inputs.

**Figure 15:**
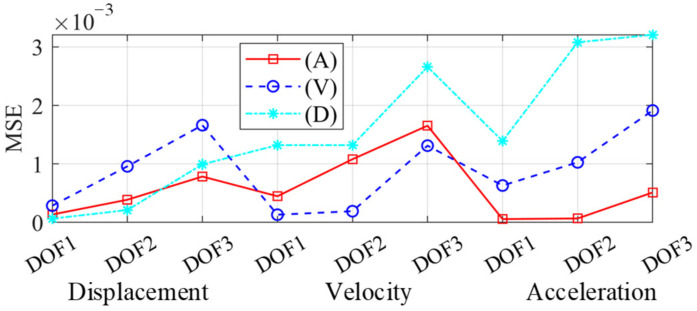
MSE with varied input data. (A: acceleration as PINN input; V: velocity as input; and D: displacement as input).

The frame response under the Kocaeli wave was used for testing. We took the velocity or displacement as the network input and re-trained the PINN. The MSEs of the responses are compared in Figure 15. Note that the MSEs of the displacement and velocity at all DOFs were scaled by ten and five respectively, for a better comparison.

When reconstructing acceleration response, the accuracy is the highest if acceleration is adopted as input. This law is universal for velocity and displacement. Since displacement and acceleration are more important for evaluating the structural state, we principally focused on their accuracy. The MSEs of displacements were close when adopting displacement or acceleration as the input, but the computed acceleration was more accurate if we adopted acceleration as the input. Therefore, the network had the best performance by adopting acceleration as the input data. Considering that accelerometers are widely used in practice, they were adopted as the data source through this study.

### 3.3. Robustness

In practice, the FEM and data inevitably have modeling errors and noise, and their influence on the PINN performance is briefly discussed here.

(1) FEM error

The PINN involves the physical loss derived from the dynamic equilibrium equation. With erroneous stiffness, damping, or mass matrices, the physical loss diverges from zero with the real responses, and this affects the PINN performance. However, when the stiffness and mass matrices are entirely scaled by an identical factor (global FEM error), the reconstructed response remains accurate. In this case, the dynamic equilibrium equation in Equation (10) was entirely scaled:

(18) $$\lambda M\overset{\cdot \cdot }{u}+\lambda C\overset{\cdot }{u}+\lambda Ku=-\lambda M1{\overset{\cdot \cdot }{u}}_{g}$$

where λ is a constant. Considering Equation (9), the overall loss function becomes:

(19) $$J=L_{T}+\lambda^{2}\beta L_{P}$$

**Figure 16:**
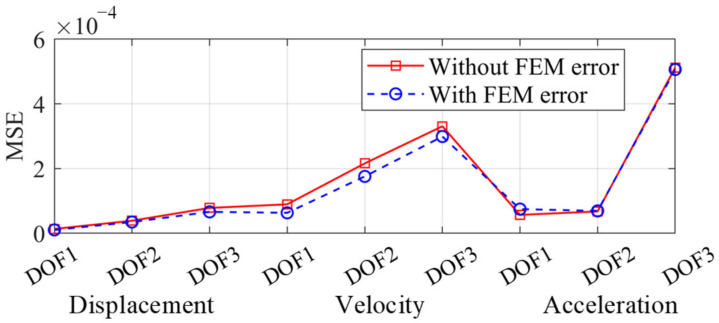
MSE distribution with and without the global FEM error.

The physical loss was scaled by λ^2^. The global FEM error is equivalent to changing the weighting factor β. When the displacement, velocity, and acceleration responses are accurate, the physical loss *L_p_* is approximate zero. *L_p_* was still approximate zero after being scaled by λ^2^. Therefore, the performance of the PINN was not seriously affected in this case. The MSEs with 20% global FEM error are shown in Figure 16, where the MSEs did not significantly increase.

With local FEM errors, the physical loss deviated from zero given the accurate responses. The physical loss *L_P_* made the displacement, velocity, and acceleration deviate from their true value to reduce *L_P_*. Therefore, the proposed framework is not robust to local FEM errors.

(2) Measurement noise

The in-field monitored data inevitably contain measurement noise, more or less. In this case, the trained network becomes seriously overfitted with a small dataset. The seismic scenario is such an example since the training dataset is extremely small. However, the physical loss can improve the networks’ robustness to the measurement noise.

**Figure 17:**
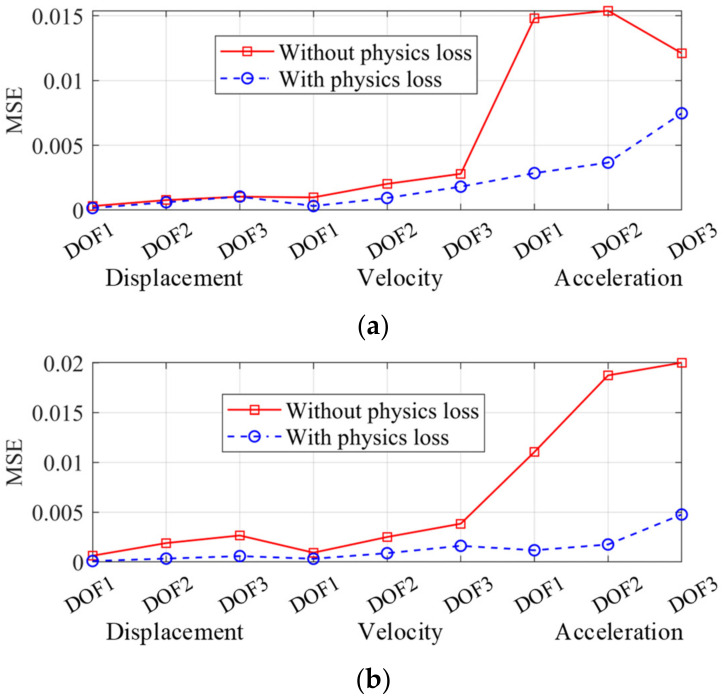
Influence of measurement noise on MSE. (**a**) Kocaeli wave; (**b**) Landers wave.

In this section, the training and testing sets are similar to the former sets. We added 10% root mean square Gaussian white noise to the input and output data when training, which made the network-learned mapping relationship overfitted. In the testing set, the network input data were noisy as well, and the ground truth was noise-free to compare with the predictions. Figure 17 shows the MSE of the reconstructed responses with and without the physical loss. The PINN-estimated responses were more accurate at all DOFs, especially for the displacements and accelerations. Therefore, adding a physical loss term into the loss function could significantly improve the NN’s robustness to measurement noise.

In this section, a three-story linear frame was adopted to validate the multi-ends autoencoder architecture and the function of the physical loss term for suppressing the overfitting effect. The details and procedures of the algorithm are explained, where both the physics-informed and ordinary neural networks can reconstruct the seismic frame response given a small training set and an extremely limited number of sensors (the accuracy of the PINN is higher). Then, the influence of the data type was analyzed to help the sensor arrangement in real applications. Finally, the robustness of the algorithm is demonstrated, and the PINN outstood with the noised data.

## 4. Further Validation of the Network’s Ability

In Section 3, we validated the network performance and the effect of the physical loss function. However, the linear assumption does not hold for all structures in real applications. The concrete material, damper, or controller can exhibit a strong nonlinear behavior. In such situations, the response reconstruction task can be much more difficult. Therefore, we further extended the proposed multi-end network model to a nonlinear example.

Since the nonlinear model is difficult to determine in real applications, the residual force in Equation (9) is unknown in most cases. Notably, if the introduced physics constraint is errored, it can even negatively affect the network’s training process. How to introduce prior physical knowledge into the network in nonlinear situations is still an open problem worthy of further studies. Nevertheless, if the adopted network model itself does not apply to the nonlinear situation, the physics loss becomes meaningless. Thus, we removed the physical loss and only trained the network with the empirical MSE loss in this section to further validate the feasibility and fitting ability of the origin network model as the basis for further introduction of any physics constraints.

**Figure 18:**
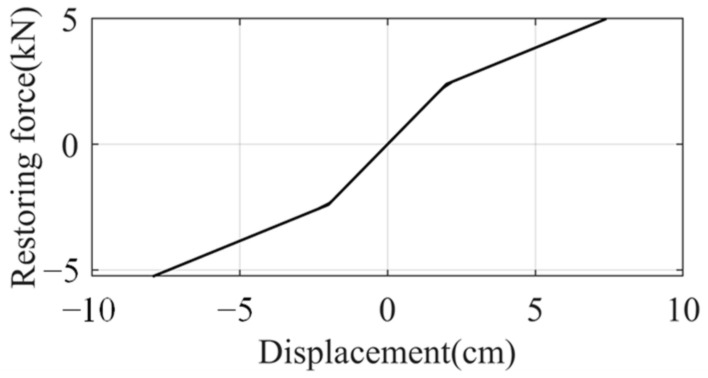
Nonlinear restoring force.

The nonlinear model is still a three-story frame [39]. The mass of each floor was 1000 kg. The initial inter-story stiffness was 120 kN/m. When the frame was in the linear scope, the natural frequencies were 0.73, 1.74, and 2.93 Hz. The damping ratios were 1.42%, 4.56%, and 5.08%. The nonlinear restoring force (see Figure 18) was adopted only on the base floor. When the inter-story displacement exceeded 2 cm, the stiffness reduced by 60%. The remaining two floors had linear stiffness. The mass, damping, and initial stiffness matrices are:

(20) M=1 × 1030001 × 103000 1 × 103 C=1.2 × 103−6 × 1020−6 × 1021.2 × 103−6 × 1020−6 × 1026 × 102 K=2.4 × 105−1.2 × 1050−1.2 × 1052.4 × 105−1.2 × 1050−1.2 × 1051.2 × 105 

The nonlinear responses are more complex than linear responses under identical loadings. The architecture in Figure 4 and hyperparameters in Table 3 were unable to learn the mapping relations among the nonlinear responses. There are two main strategies to improve the network learning ability: (1) add more layers; (2) increase the kernel numbers at each layer. In this section, the second strategy was adopted. The kernel numbers of all convolutional and deconvolutional layers were multiplied by a factor F (F = 1~6) to analyze the influence of the kernel number. When F = 1, the network was exactly the architecture described in Table 3, which served as a baseline.

**Figure 19:**
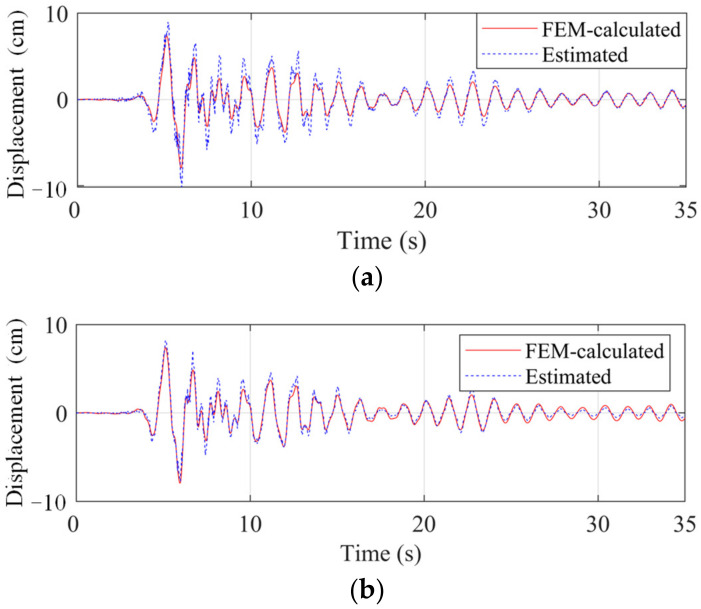
Reconstructed displacement at the base floor with varied kernel numbers. (**a**) F = 1; (**b**) F = 6.

The training set was the nonlinear seismic responses under the first sixth seismic waves in Table 2. The test set contained the nonlinear responses under the Landers and Kocaeli waves. The amplitudes of the seismic waves were adjusted to provide that the maximum displacement of the base floor can exceed 2 cm to trigger the nonlinearity. The reconstructed displacement (under the Kocaeli wave) at the base floor is shown in Figure 19.

When F = 1, the reconstructed high-frequency components deviated from the ground truth. When F = 6, the high-frequency responses became more accurate. This phenomenon is easy to understand. When the response involves multi-frequency components, the component with a greater value is first learned. For the studied structure, the low-frequency component dominates the response and has a greater value. Therefore, the network (F = 1) first learns the low-frequency mapping relationships. With the improvement of the network complexity (F = 6), the network has more kernels and parameters to learn the high-frequency mapping relations.

**Figure 20:**
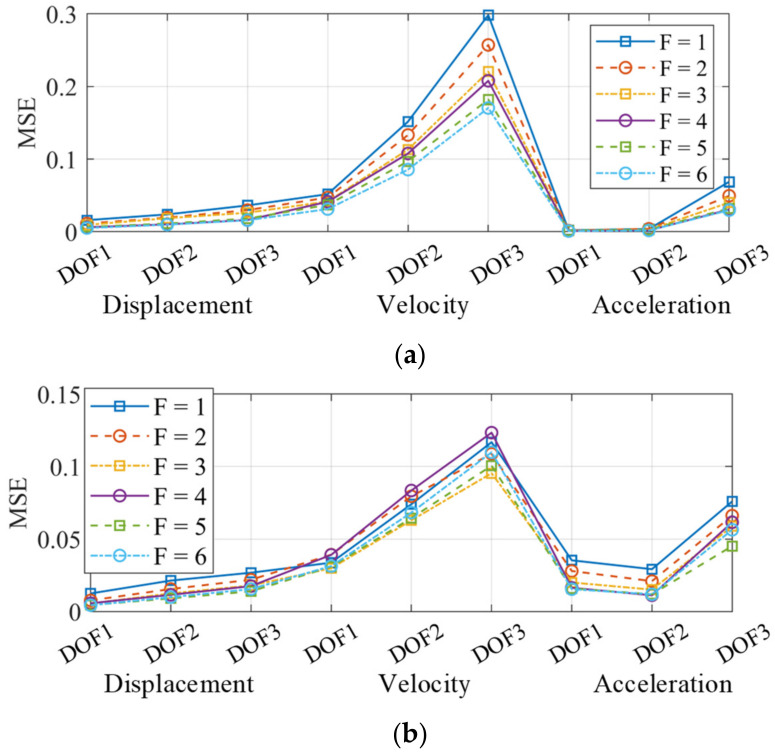
MSE of all reconstructed responses. (**a**) Landers wave; (**b**) Kocaeli wave.

The responses’ MSEs at all DOFs are shown in Figure 20. The MSE distribution in Figure 20a can better illustrate the influence of the kernel number. With a greater F, the MSEs of all responses decreased. When F increased from one to six, the kernel and parameter numbers multiplied by six times, which could lead to a more serious overfitting effect given an identical training dataset. However, the MSEs did not become worse, which indicated that the positive influence overwhelmed the negative influence when increasing the network complexity. In Figure 20b, the MSEs of displacement and acceleration still decreased with a greater F. However, the MSE of velocity did not monotonously decrease and it only has a decreasing trend. This is partly because the mapping from acceleration to velocity is more complex (the phase difference between velocity and acceleration is π/2, rather than π).

Except for the network width (i.e., the kernel numbers), the network depth, kernel sizes, skip connections, and bottleneck size can all influence the fitting ability of the network in a non-monotonic way [40]. Herein, we only focused on the width of the network, as increasing the width is a relatively simple way to strengthen the network’s learning ability. To study the influences of other factors, one should run more numerical tests.

This section adopted a nonlinear system for demonstrating the applicability of the multi-ends NN. Since the nonlinear seismic response is more complex than the linear response, the required network became more complex. Even so, the proposed multi-end architecture could still predict the nonlinear response given a small training set without the physical loss.

Section 3 and Section 4 separately adopted linear and nonlinear numerical models to validate the applicability of the multi-end network for predicting the responses of multi-DOF systems. In the linear part, the restoring, damping, and inertial forces were computed and the residual force-based physical loss could prevent the training from overfitting given a small training set. In the nonlinear system, since the nonlinear model and restoring force is usually unknown, the multi-end autoencoder network was trained without physical loss, but it also provided sound reconstruction results. In conclusion, the multi-end autoencoder could learn to map the linear or nonlinear relationships between various responses at different DOFs, which can help to estimate the complete seismic responses. When the physical knowledge of the structural parameters is clear, it can be transformed and added into the loss function to improve the network performance on small training sets. If the physical information is incomplete, the proposed network architecture can still achieve the response reconstruction task with high accuracy without physical constraints, which is also the prerequisite for further studying the effect of prior physical knowledge in nonlinear situations. One limitation of this paper is that it only involved numerical validation, but no experiment was conducted. However, the numerical simulations still witnessed the superiority of the multi-end autoencoder and the effectiveness of the PINN.

## 5. Conclusions

This paper adopted a multi-end convolutional autoencoder to reconstruct the complete seismic response of the multi-DOF systems. Additional physical information was integrated into the loss function to guide the training process. Through two numerical simulations, the following conclusions were drawn:

(1) The proposed framework could reconstruct the seismic response of multi-DOF systems given a small training dataset. Theoretical basis explained that the encoder can extract the shared features and reduce the parameter number as well as training costs. The decoder could map the extracted features to the target responses, where the skip connections were essential to retaining the completeness of the information.

(2) In the linear example, the dynamic equilibrium proved to be a good physical constraint to modify the reconstructed responses with a small training dataset. The prior knowledge-based constraint was equivalent to a regularization term that can suppress the overfitting effect and improve the generalization ability, especially when the training data are noised. In addition, the proposed framework was validated to be robust to global modeling errors.

(3) The proposed multi-end autoencoder applied to both linear and nonlinear systems. If the studied structure is associated with nonlinearity, a more sophisticated network is necessary, i.e., the network should involve more learnable parameters.

## Figures and Tables

**Table 1:** Summary of the physics-informed neural network applications.

No.	Title 2	Hybrid Mode	Training Data	Neural Type
[17,18]	PDE	Loss function	Y	DNN
[19]	PDE	Loss function	N	CNN
[20]	PDE	Loss function	N	CNN
[21]	PDE	Loss function	N	CNN
[22]	PDE	Loss function	Y	DNN
[15]	Hydrodynamic	Loss function	N	DNN
[25]	Geology (seismic wave equation)	Loss function	Y	DNN
[28]	System identification	Loss function	Y	DNN
[33]	Subsurface flow	Loss function	Y	DNN
[30]	Seismic analysis	Loss function	Y	CNN
[34]	Koopman decompositions	Loss function	Y	RNN
[16]	Fatigue-corrosion analysis	Physics-based network design	Y	RNN
[26]	Fatigue of bearing	Physics-based network design	Y	RNN
[27]	Power system	Physics-based network design	Y	DNN
[29]	System control	Physics-based network design	Y	DNN
[31]	Geology	Physics-based network design	Y	LSTM
[32]	SHM	Loss and physics-based network design	Y	DNN
[23]	Hydrodynamic and discrete element	Loss function in both hidden and Output layers AND physics-based network design	Y	CNN

**Table 2:** The seismic waves’ information.

Number	Name	Frequency Range (Hz)	Record Length (s)
1	Chichi	0.02–50.0	52.78
2	Friuli	0.1–30.0	36.32
3	Northridge	0.12–23.0	39.88
4	Trinidad	0.15–30.0	21.4
5	Imperial Valley	0.1–40.0	39.48
6	Kobe	0.1–unknown	40.9
7	Kocaeli	0.07–50.0	34.96
8	Landers	0.08–60.0	48.09

**Table 3:** The details of the network.

Layer	Kernel Number	Kernel Size	Stride Size	Padding	Input Shape	Output Shape
**Encoder**						
Conv1	128	3 × 2	1 × 2	same	3 × 400 × 1	3 × 200 × 128
Conv2	64	3 × 2	1 × 2	same	3 × 200 × 128	3 × 100 × 64
Conv3	32	3 × 2	1 × 2	same	3 × 100 × 64	3 × 50 × 32
Conv4	16	3 × 2	1 × 2	same	3 × 50 × 32	3 × 25 × 16
**Decoder**						
Deconv1	32	3 × 2	1 × 2	same	3 × 25 × 16	3 × 50 × 32
Deconv2	64	3 × 2	1 × 2	same	3 × 50 × 32	3 × 100 × 64
Deconv3	128	3 × 2	1 × 2	same	3 × 100 × 64	3 × 200 × 128
Deconv4	1	3 × 2	1× 2	same	3 × 200 × 128	3 × 400 × 1
**Skip connection** (Deconvolutional layers)						
Skip1	128	3 × 2	1 × 2	same	3 × 200 × 128	3 × 200 × 128
Skip2	64	3 × 2	1 × 2	same	3 × 100 × 64	3 × 100 × 64
Skip3	32	3 × 2	1 × 2	same	3 × 50 × 32	3 × 50 × 32

Note: the dimension indexes indicate height × width (×depth), and the layer numbers increase with the depth in the network.

## Data Availability

Not applicable.

## Abbreviations

The following abbreviations are used in this manuscript:

SHM	structural health monitoring
NN	neural network
DNN	deep neural network
FEM	finite element model
PDE	partial differential equation
PIDL	physics-informed deep learning
CNN	convolutional neural network
RNN	recurrent neural network
LSTM	long short-term memory
PINN	physics-informed neural network
DOF	degrees of freedom
MSE	mean square error
